# Precision Cardiogenomics in Athletes

**DOI:** 10.3390/ijms27125250

**Published:** 2026-06-10

**Authors:** Pari Goyal, Alwaleed Aljohar, Reid A. Mitchell, Nathaniel Moulson, James McKinney, Saul Isserow, Zachary Laksman

**Affiliations:** 1 SportsCardiologyBC, The University of British Columbia Hospital, Vancouver, BC V6T 2B5, Canada; parig06@student.ubc.ca (P.G.); alwaleed.aljohar@vch.ca (A.A.); reid.mitchell@vch.ca (R.A.M.); nathaniel.moulson2@vch.ca (N.M.); james.mckinney@ubc.ca (J.M.); saul.isserow@vch.ca (S.I.); 2 National Center for Sudden Death, King Saud University Medical City, Riyadh 11472, Saudi Arabia; 3 Division of Cardiology, Department of Medicine, University of British Columbia, Vancouver, BC V5Z 1M9, Canada

**Keywords:** sudden cardiac death, athletes, exercise, genomics, cardiomyopathies, channelopathies

## Abstract

Sudden cardiac death (SCD) in athletes often represents the first manifestation of an underlying inherited cardiovascular disorder exposed by adrenergic stress, altered calcium cycling, mechanical loading, and metabolic demand during intense exercise. This review focuses on the molecular architecture that links genotype to arrhythmogenic phenotype in athletes, emphasizing sarcomeric force generation and energetic inefficiency in hypertrophic cardiomyopathy, desmosomal failure and Hippo/Wnt/transforming growth factor-beta (TGF-β) signaling in arrhythmogenic cardiomyopathy, and ion-channel and calcium/calmodulin-dependent protein kinase II (CaMKII)calcium handling abnormalities in inherited channelopathies. This review further examines how exercise-induced physiological remodeling intersects with these pathways through insulin-like growth factor-1 (IGF-1)/phosphoinositide 3-kinase (PI3K)/ protein kinase B (AKT) signaling, mitochondrial biogenesis, oxidative stress, inflammatory signaling, and epigenetic regulation. Attention is given to the molecular basis of genotype-positive/phenotype-negative states, variable penetrance, and exercise-mediated disease expression. Finally, the integration of molecular biology with genomic data, polygenic risk, and emerging digital phenotyping is discussed to refine mechanism-based risk stratification and identify future therapeutic targets for prevention of SCD in athletes.

## 1. Introduction

Sudden cardiac death (SCD) is the leading medical cause of death in athletes under the age of 35 years [1]. The impact of SCD is profound and is often the first manifestation of underlying cardiac disease in apparently healthy, asymptomatic individuals [2]. In the general population, SCD occurs in approximately 1.3 per 100,000 individuals [3]. However, this risk can be magnified in young athletic populations, estimated at 1 per 66,000 to 88,000, with even higher incidences in specific populations such as elite male soccer and basketball players [4,5,6]. During exercise, heart rate (HR) increases through a rapid withdrawal of parasympathetic nervous system (PNS) activity, followed by progressive sympathetic nervous system (SNS) activation [7]. These rapid shifts in autonomic balance may increase myocardial excitability, promote electrical instability, and lower the threshold for ventricular arrhythmias in individuals with underlying cardiac disease. Consequently, these events often occur during or immediately after intense physical activity, suggesting that exertion may act as a trigger for individuals with underlying genetic predispositions to cardiac vulnerabilities [8,9]. Importantly, the transition from peak exertion to early recovery is characterized by persistent catecholaminergic drive, dynamic shifts in repolarization reserve, and transient calcium handling instability, creating a vulnerable electrophysiological window in genetically susceptible myocardium.

The pathophysiology underlying SCD in athletes remains incompletely understood, which limits opportunities for tailored therapies and accurate risk stratification. Although clinical syndromes associated with SCD are increasingly recognized and detected earlier, the molecular pathways underlying disease penetrance, trigger sensitivity, and exercise-dependent disease progression have yet to be fully elucidated. A more mechanistic framework is needed because pathogenic variants do not act in isolation; rather, they perturb force generation, intercellular adhesion, calcium homeostasis, mechano-transduction, inflammatory signaling, and cellular energetics, which are then modulated by exercise intensity and training load [9,10,11].

The specific contribution of this review is to synthesize these disease-specific studies into a pathway-centered framework for precision cardiogenomics in athletes. Rather than reviewing each condition solely as an isolated clinical diagnosis, we emphasize how sarcomeric dysfunction, desmosomal instability, ion channel dysfunction, calcium handling abnormalities, mechano-transduction, inflammation, metabolic stress, and exercise exposure interact to shape penetrance and arrhythmic risk. This framework is intended to complement existing sports cardiology and inherited arrhythmia reviews by linking molecular pathogenesis to clinically relevant questions in athlete evaluation, shared decision-making, longitudinal monitoring, and future AI-enabled risk stratification.

### Methods: Literation Search and Source Selection

This article was conducted as a narrative review designed to synthesize mechanistic, genetic, and translational literature relevant to sudden cardiac death risk in athletes. We searched PubMed/MEDLINE, Google Scholar, and major cardiology society guideline documents for English language publications from January 2000 to May 2026. Priority was given to major society statements, clinical guidelines, prospective human studies, genotype–phenotype cohort studies, mechanistic reviews, and high-impact experimental studies addressing inherited cardiomyopathies, inherited channelopathies, exercise-induced cardiac remodeling, sports participation, polygenic risk, wearable monitoring, and artificial intelligence in cardiovascular risk stratification.

Search terms included combinations of: “sudden cardiac death,” “athletes,” “sports cardiology,” “hypertrophic cardiomyopathy,” “arrhythmogenic cardiomyopathy,” “arrhythmogenic right ventricular cardiomyopathy,” “long QT syndrome,” “catecholaminergic polymorphic ventricular tachycardia,” “Brugada syndrome,” “short QT syndrome,” “exercise,” “genotype positive phenotype negative,” “polygenic risk score,” “wearable ECG,” “artificial intelligence,” “cardiogenomics,” “calcium handling,” “CaMKII,” “desmosome,” “Wnt beta-catenin,” “Hippo signaling,” “TGF-beta,” and “inflammasome”. Additional references were identified from the bibliographies of major reviews and guidelines.

Studies were included if they addressed: (1) inherited cardiovascular disorders associated with sudden cardiac death in athletes; (2) molecular or genetic mechanisms linking genotype to phenotype; (3) exercise as a modifier of arrhythmic or structural disease expression; (4) translational tools such as genetic testing, polygenic risk scores, wearables, or AI-based risk stratification. Studies were excluded if they focused exclusively on non-athlete populations without relevance to exercise physiology, were not available in English, lacked peer review, or addressed acquired cardiovascular disease without a clear genetic or exercise-related component. Because this was a narrative review, no formal meta-analysis or quantitative risk-of-bias scoring was performed; however, the revised manuscript explicitly distinguishes human clinical evidence, observational cohort data, guideline-based recommendations, and preclinical or hypothesis-generating mechanistic evidence.

## 2. Physiological and Molecular Responses to Exercise

The role of exercise in modulating SCD risk is dose-dependent, with diminishing health benefits at sustained, higher intensities. Regular physical activity promotes cardiovascular health, enhances myocardial efficiency, and improves metabolic function [12,13]. However, long-term exposure to high-intensity training may result in significant chamber dilatation or accelerated atherosclerosis, subjecting athletes to risks of developing atrial fibrillation, coronary artery disease, or unmasking occult arrhythmias and cardiomyopathies [14,15].

Physiological remodeling is largely mediated by the activation of insulin-like growth factor-1 (IGF-1) signaling and downstream phosphoinositide 3-kinase/ protein kinase B (PI3K/AKT) pathways, both of which are central regulators of exercise-induced cardiac growth [16]. Mechanical stress against myocardial cells and an increased stroke volume during exercise stimulate IGF-1 release and receptor activation. This leads to cardiomyocyte growth, angiogenesis, improved excitation–contraction coupling, and anti-fibrotic signaling. This pathway can also support proportional hypertrophy with preserved systolic and diastolic function [16].

Mitochondrial function also represents a key interaction area between exercise, adaptation, and disease progression. During exercise, increases in heart rate and contractility require more ATP, and this demand is matched by enhanced mitochondrial biogenesis, oxidative capacity, and substrate flexibility [17]. Additionally, sympathetic activation increases intracellular calcium, which enters the mitochondria and stimulates key enzymes of the Krebs cycle to increase ATP production, supporting acute increases in workload [18].

While these changes are physiological, they often have a degree of clinical overlap with features of inherited cardiomyopathies [19]. For example, elite athletes may have left ventricular hypertrophy reaching a range that may mimic HCM [20]. Physiological remodeling and cardiac pathologies may phenotypically overlap; however, their underlying molecular upstream drivers are different, including signaling cascades, stress-activated pathways, fibrosis-promoting signaling, and energetic regulation. One example includes adaptive IGF-1/PI3K signaling versus disease-associated sarcomere dysfunction [16]. In physiological remodeling, IGF-1/PI3K/AKT signaling supports proportional myocyte growth, preserved capillary density, mitochondrial adaptation, and anti-fibrotic transcriptional programs. In contrast, pathological hypertrophy in inherited cardiomyopathy is more closely linked to sarcomere stress, oxidative stress, energetic inefficiency, and extracellular matrix expansion [16]. Furthermore, HCM is also characterized by myocardial angiogenesis, which plays a key role in maintaining adequate nutrient supply, to avoid myocardial dysfunction. Hypoxia-inducible factor 1*α* (HIF1*α*) is a major transcription factor that regulates oxygen homeostasis through angiogenesis. In physiological adaptations, induced HIF1*α* promotes cardiomyocyte growth and angiogenesis; however, in pathological hypertrophy, there is a degradation of HIF1*α*. This leads to an imbalance between myocardial growth and capillary density, thereby promoting maladaptive hypertrophy onset [21].

Endurance training has been shown to enhance the secretion of the mitochondrial open reading frame of 12S rRNA-c (MOTS-c) from skeletal muscle, and may enhance mitochondrial respiration by activating the AMP-activated protein kinase (AMPK)/PGC-1a pathway [22]. These pathways coordinate the cellular response to energetic stress by sensing reduced ATP during exercise, and activating alternative pathways that increase mitochondrial biogenesis, oxidative capacity, and metabolic efficiency [23].

Exaggerated SNS activation and reduced vagal tone as a result of exercise are also recognized as contributors to arrhythmic risk and SCD in athletes [8,9]. In underlying cardiovascular disease states, persistent SNS overactivity is often accompanied by the activation of the renin–angiotensin–aldosterone system (RAAS), which increases vascular resistance and myocardial load [24]. Many athletic disciplines are characterized by brief but intense bursts of physical exertion combined with high levels of psychological stress, such as sprinting events, gymnastics, power sports, basketball, soccer, and tackle football [4,25,26]. Collectively, these diverse genetic conditions predispose young athletes to life-threatening events under physical stress.

**Figure 1:**
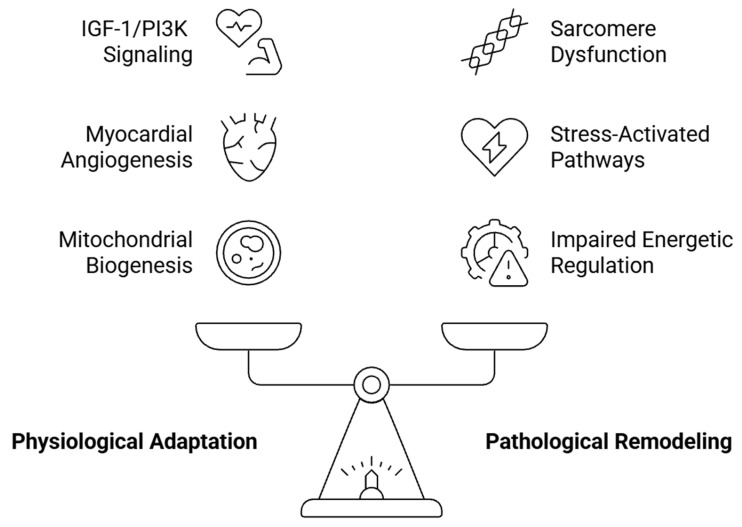
Physiological Adaptations and Pathological Remodeling in Response to Physical Activity. Balance between the cardiovascular benefits of regular exercise and the potential structural, electrical, and clinical risks associated with high-intensity training, especially for athletes predisposed to genetic cardiovascular diseases.

Differentiating between physiological adaptation and early disease expression through clinical testing and imaging can be challenging. Clinical tools such as genetic testing, imaging, and exercise testing aim to distinguish physiological adaptations from early pathological changes. Physiological and pathological remodeling may share some superficial imaging features; however, their upstream molecular programs differ substantially. Athlete’s heart is characterized by coordinated growth signaling, mitochondrial adaptation, and preserved mechano-energetic efficiency, whereas inherited cardiomyopathy reflects mutation-driven defects in sarcomere function, desmosomal integrity, ion channel behavior, or cytoskeletal signaling, with secondary activation of fibrosis and inflammatory pathways [17,21,23,24] (Figure 1).

## 3. SCD in Athletes: Mutations and Molecular Pathways

**Figure 2:**
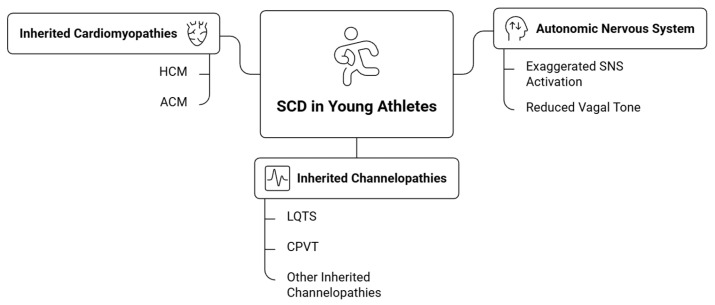
Common Inherited Causes of Sudden Cardiac Death (SCD) in Young Athletes. HCM—hypertrophic cardiomyopathy, ACM—arrhythmogenic cardiomyopathy, CPVT—catecholaminergic polymorphic ventricular tachycardia, LQTS—long QT syndrome, other inherited channelopathies, and the autonomic nervous system.

Inherited cardiomyopathies and channelopathies that lead to structural or electrical abnormalities are common causes of SCD in athletes [4,27]. These include diseases such as hypertrophic cardiomyopathy (HCM), arrhythmogenic cardiomyopathy (ACM), catecholaminergic polymorphic ventricular tachycardia (CPVT), long QT syndrome (LQTS), and other inherited channelopathies [28] (Figure 2, Table 1). Many of these conditions follow an autosomal dominant inheritance pattern. Therefore, first-degree relatives of affected individuals carry a 50% risk of inheriting the mutated genes [29].

### 3.1. HCM

HCM is a recognized cause of SCD in athletes and is fundamentally a disease of sarcomeric force generation and mechano-energetic coupling. It is characterized by dysfunctional myocardial hypertrophy, even in the absence of abnormal loading conditions, and often accompanied by myocyte disarray, diastolic dysfunction, and increased arrhythmogenic risk [30].

At the molecular level, the majority of pathogenic variants in HCM occur in genes encoding sarcomeric or sarcomere-associated proteins, particularly *MYH7* and *MYBPC3*, encoding β-myosin heavy chain and myosin-binding protein C, respectively. These proteins are integral in thick filament structures, and mutated variants can either reduce or enhance motor cross-bridge kinetics. Mutations in thin filament regulator proteins, such as Troponin T (*TNNT2*), troponin I (*TNNI3*), and tropomyosin (*TPM1*), also result in increased calcium sensitivity. These variants can also produce a state of hypercontractility and lead to impaired energy homeostasis and relative ATP depletion. Additional contributors to HCM include mutations in genes encoding Z-disk and nonsarcomeric proteins. The Z-disk is importantly implicated in sensing and transducing biomechanical stress, and impaired stress sensing can lead to an excessive hypertrophic response to mechanical load. These primary responses activate downstream pro-hypertrophic signaling cascades, including calcium-dependent pathways and mitogen-activated protein kinase (MAPK) signaling, which promote pathological cardiac remolding [31,32].

During intense exercise, increased contractility demands can exacerbate dysfunction of the actin–myosin interactions and lead to an increased likelihood of ventricular arrhythmias [33]. In this context, exercise also has the potential to amplify pre-existing sarcomere inefficiency and calcium-dependent afterdepolarization risk, thereby coupling genotype to exertional ventricular arrhythmogenesis [33].

### 3.2. ACM

ACM is an inherited myocardial disease characterized by progressive fibrofatty replacement of ventricular myocardium and a high risk of ventricular arrythmias and SCD. Initially considered a predominantly right ventricular disease, it is now recognized to affect the left ventricle, either in isolation or alongside the right ventricle. At the cellular level, ACM is most commonly caused by variants of the intercalated disk, where desmosome proteins function as important mechanosensors and mechanical junctions that maintain cardiomyocyte integrity [34].

The main function of the cardiac desmosomal proteins is to mediate cell-to-cell adhesion and cytoskeletal coupling within the intercalated disk, and those include plakophlin-2 (*PKP2*), desmoplakin (*DSP*), plakoglobin (*JUP*), desmoglein-2 (*DSG2*), and desmocollin-2 (*DSC2*). These proteins form a structural network that anchor intermediate filaments, such as desmins, between adjacent cardiomyocytes, allowing for coordinate force transmission and mechanical resilience [34,35]. Disruption of desmosome adherence junctions due to increased mechanical stress can lead to the dissociation of plakoglobin from desmosomes and result in unstable intercalated disks [35].

ACM demonstrates strong evidence for exercise-aggravated disease expression, with high-intensity endurance training affecting right ventricular remodeling, arrhythmic events, and heart failure progression [6]. Desmosomal failure activates Hippo signaling, suppressing canonical Wnt/β-catenin signaling. Instability in the intercalated disks displaces β-catenin from the cell membrane, and it fails to translocate to the nucleus where it modifies transcriptional activity. This suppression of the canonical Wnt/β-catenin pathway leads to a loss of myocyte-specific gene expression [36]. The resulting remodeling involves fibrofatty replacement of myocardial tissue with adipose tissue and collagen. This change creates electrical discontinuity as fat is non-conductive, creating a physical barrier that slows conduction of the electrical signal [37]. Parallel activation of transforming growth factor-beta (TGF-β) signaling drives myofibroblast activation and extracellular matrix deposition. In addition, disruption of the intercalated disk complex perturbs sodium channel organization and gap–junction homeostasis, creating early electrical phenotypes that may precede overt structural disease. Together, these coupled defects provide a mechanistic basis for exercise-accelerated fibrofatty replacement and ventricular arrhythmias [38].

### 3.3. LQTS

LQTS is an inherited channelopathy marked by QT interval prolongation on ECG readings, reflecting delayed ventricular repolarization. Affected individuals may experience syncope, polymorphic ventricular tachycardia (Torsade de Pointes), or even SCD, particularly triggered by exertion, adrenaline surges, and stress [10]. Genotype–phenotype correlations are observed in different gene specific triggers. LQT1 cardiac events frequently occur during exercise, LQT2 during instantaneous emotional or auditory stress, and LQT3 most often at rest [39].

This condition may arise from multiple genes that encode for different cardiac ion channels, but the three major subtypes of genes involve *KCNQ1* (LQT1) and *KCNH2* (LQT2), which encode potassium channels responsible for repolarizing outward currents, and *SCN5A* (LQT3), which encodes the cardiac sodium channel responsible for inward depolarizing currents. Loss-of-function mutation in potassium channels or gain-of-function mutations in sodium channels prolong the action potential duration, thereby extending repolarization and increasing susceptibility to arrhythmias [40].

During exercise, β-adrenergic stimulation increases heart rate and enhances repolarizing potassium currents, particularly the slow delayed rectifier current (IKs), to shorten the cardiac action potential and allow adequate diastolic filling time. Times of rapid heart rate and oxidative stress can exacerbate repolarization abnormalities, especially in LQT1, where impaired IKs current limits the heart’s ability to adapt to increased load. Individuals with malfunctioning IKs channels are less effective at shortening their QT intervals compared to normal individuals, and this increases the probability of Torsade de Pointes, a rare, life-threatening form of polymorphic ventricular tachycardia [41].

### 3.4. CPVT

CPVT is an inherited arrhythmia syndrome characterized by exercise- or emotion-induced ventricular tachyarrhythmias occurring in the absence of a structural heart disease. This disease onset typically manifests during childhood, presenting itself as exercise-induced or emotionally triggered syncope or SCD. These stressors increase sympathetic tone and exacerbate underlying defects in intracellular calcium handling [42].

At the molecular level, CPVT is fundamentally a disease of intracellular calcium homeostasis. The majority of cases are caused by mutations in the ryanodine receptor 2 (*RYR2*) gene. This gene encodes the cardiac ryanodine receptor, which is a calcium release channel located on the sarcoplasmic reticulum (SR) that mediates calcium-induced calcium release during excitation–contraction coupling. Another cause of CPVT includes variants of the cardiac calsequestrin 2 (*CASQ2*) gene which encodes a key SR calcium-binding protein that regulates calcium storage and release. Additional rare variants have been identified in genes encoding calcium handling proteins, including triadin (*TRDN*) and calmodulin 1, 2, and 3, (*CALM1*, *CALM2*, *CALM3*), further highlighting the role of calcium homeostasis [43].

Pathogenic variants in these genes destabilize the RYR2 complex, resulting in diastolic calcium leak from the SR, particularly under conditions of β-adrenergic stimulation. Elevated levels of cytosolic calcium activate the sodium–calcium exchanger and generate an inward current of sodium ions. This has the potential to produce delayed afterdepolarizations (DADs), and if large enough, these DADs can trigger premature action potentials resulting in arrhythmias [43]. Additionally, activation of β-adrenergic signaling leads to the activation of the calcium/calmodulin-dependent protein kinase II (CaMKII) sensor. Overactive CaMKII phosphorylates RYR2 and sodium channels in cardiomyocytes, disrupting the precise balance of excitation–contraction coupling required for high-output cardiac performance [44]. The remodeling phase involves dysfunctional RYR2 receptors that allow for spontaneous calcium release during the heart’s resting phase. This results in delayed depolarizations, and this dysfunction can act as a pro-arrhythmic trigger, leading to spontaneous electrical pulses [45].

### 3.5. Other Channelopathies

Other inherited channelopathies include Brugada Syndrome and Short QT Syndrome, which are also known causes of SCD in young, structurally normal hearts. However, in contrast to LQTS and CPVT, these conditions are less associated with exertion-triggered arrhythmic events and therefore play a more limited role in athlete-specific SCD.

Brugada syndrome is an inherited channelopathy characterized by a pattern of coved ST-segment elevation with T-wave inversions in the right precordial leads and an increased risk of ventricular fibrillation. Arrhythmic events in Brugada syndrome typically occur at rest, during sleep, or in the setting of increased vagal tone (slow heart rate, post-prandial state), rather than during exercise, and common triggers include fever, sodium channel-blocking medications, and autonomic imbalance [46,47].

Short QT Syndrome (SQTS), in contrast, is a rare but highly malignant and fatal channelopathy defined by abnormally shortened ventricular repolarizations and a reduced QT interval. The shortening of the repolarization reduces the refractory period and promotes arrhythmias by increasing the dispersion of repolarization across the myocardium [48]. Similar to Brugada syndrome, some sporadic observations have confirmed that slowing heart rate has been accompanied by shortening of the QT interval, resulting in SQTS during bradycardia rather than highly exertional activity [49].

## 4. Physiological Impacts of Exercise on Underlying Cardiac Conditions

**Figure 3:**
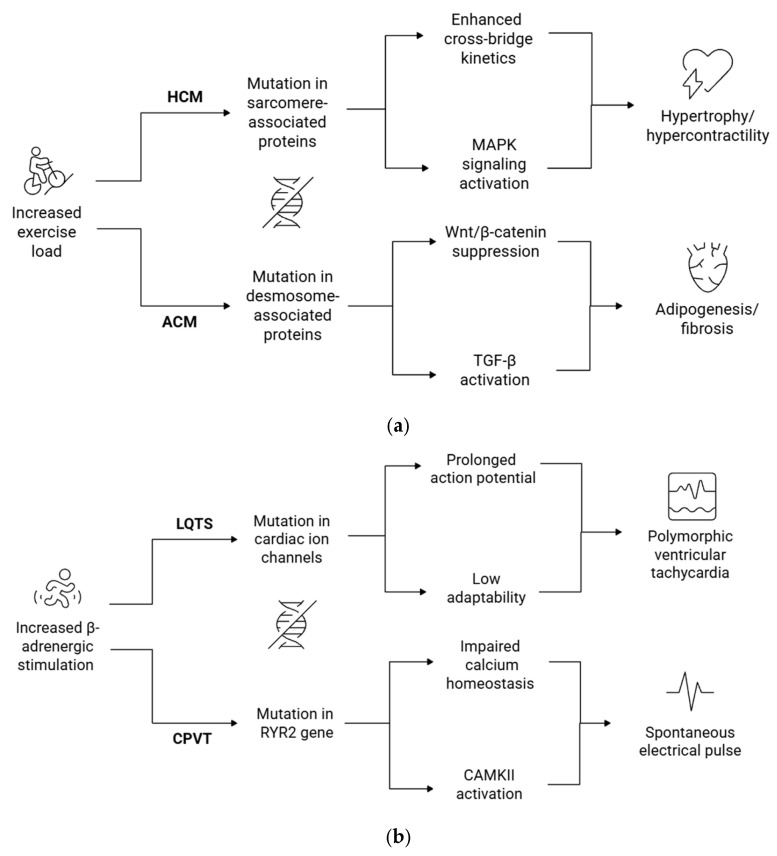
(**a**) Cardiomyopathy Mechano-transduction Map. Increased exercise load in individuals with specific gene mutation in sarcomere- or desmosome-associated proteins can cause disruptions in signaling pathways, including enhanced cross-bridge cycling, mitogen-activated protein kinase (MAPK) signaling, Wnt/β-catenin suppression, or transforming growth factor-beta (TGF-β) activation. These pathways trigger pathological remodeling of the heart, promoting hypertrophy, hypercontractility, adipogenesis, and fibrosis in the myocardium. (**b**) Channelopathy Mechano-transduction Map. Increased β-adrenergic stimulation, due to high-intensity exercise or stress, in individuals with mutated cardiac ions channels or ryanodine receptor 2 (RYR2), can lead to maladaptive cascade signaling. These pathways can trigger prolonged action potentials, low adaptability to load, impaired calcium handling, and calcium/calmodulin-dependent protein kinase II (CAMKII) activation, and together, these molecular changes contribute to spontaneous electrical pulses, and even more severe arrhythmias.

There is a complex relationship between exercise and inherited cardiac conditions. Approximately 80% of non-traumatic SCDs in young athletes are a result of inherited cardiac abnormalities, including HCM, ACM, LQTS, and CPVT, highlighting the importance of early detection and surveillance in these individuals [45] (Figure 3a,b). High-intensity or endurance exercise can be a potential upstream trigger for individuals with certain genetic predispositions, particularly those affecting myocardial structure or ion channel function, leading to arrhythmias and even SCD [9]. Symptoms such as exertional syncope, palpitations, or chest pain require cardiac consultation with potential investigation consisting of cardiac imaging, ECG monitoring, and exercise testing.

### 4.1. Autonomic Nervous System

Physical exertion activates the sympathetic nervous system and produces acute adrenaline surges. This trigger activates the β-adrenergic signaling cascade, increasing intracellular cyclic AMP and calcium influx. Elevated intracellular calcium binds to calmodulin and activates CaMKII, a multifunctional kinase essential for the sympathetic response. High levels of calcium allow CaMKII autophosphorylation and persistent activation. This pathway can result in sarcoplasmic protein excitation–contraction coupling and sustained tachycardia during or after exercise. Excessive CaMKII activity can also destabilize calcium handling, increasing the spatial dispersion of repolarization [50]. This pathway is particularly relevant in genotype-specific channelopathies, such as LQT1, in which adrenergic stimulation increases arrhythmic risk, providing a mechanistic bridge between exercise and triggered molecular pathways [51].

### 4.2. Inflammatory Pathways

Across both inherited cardiomyopathies and exercise-induced phenotypes, evidence supports a convergent inflammasome–fibrosis axis as a potential shared final common pathway of myocardial remodeling. Diverse upstream triggers, including desmosome dysfunction, sarcomere stress, cytoskeletal abnormalities, and intense exercise-induced oxidative stress can promote the activation of inflammatory signaling complexes such as the NLRP3 inflammasome [52,53]. The innate immune complex of the NLRP3 inflammasome assembles cardiomyocytes in response to mechanical stress and reactive oxygen species (ROS), triggering an internal inflammatory cascade. This signaling can also contribute to cytokine release, and can ultimately cause atrial fibrosis, hypertrophy, or cellular apoptosis [52]. While adaptive inflammation is important, persistent inflammation due to mechanical stress from intense physical activity can promote fibro-inflammatory remodeling through cytokine and TGF-β mediated signaling, resulting in fibroblast activation and differentiation in the matrix [54]. Wnt and TGF-β signaling pathways can further interact within this regulatory feedback loop that influences fibroblast and scar formation [54].

Emerging evidence also suggests that circulating microRNAs (miRNAs) and inflammasome signaling can also function as molecular messengers, and link training load to electrical vulnerability. Mechanical stress and cell injury from exercise can alter circulating RNA, which regulates gene expression through post-transcriptional silencing of target mRNAs [55]. These altered miRNAs can influence pathways involved in hypertrophy, angiogenesis, fibrosis, and metabolic adaptations, and can also be used as biomarkers of cardiovascular remodeling.

Given the heterogeneity in molecular drivers of disease progression, exercise-related risks do not apply uniformly across all cardiovascular pathological conditions [56]. The framework for athlete participation in sports is shifting, and even those positive for pathogenic variants may not require restriction from sports. A useful unifying framework could be a ROS–CaMKII–inflammasome axis which can help explain how intense exercise, when superimposed on inherited defects in calcium handling or mechano-transduction, increases oxidative stress, sustains pathological CaMKII activation, and promotes NLRP3-dependent fibro-inflammatory remodeling.

### 4.3. Mitochondrial and Metabolic Signaling

Mitochondrial and metabolic signaling is another pathway that modulates the response to exercise stress. In a healthy heart, approximately 70% of ATP is generated through mitochondrial fatty acid oxidation, with the remainder derived from glucose oxidation. However, during periods of increased workload such as exercise, the heart demonstrates metabolic flexibility, with a relative shift towards higher glucose utilization to provide ATP [18]. Short-term increases in glucose oxidation represent an adaptive metabolic reserve to support acute stressors; however, persistent reliance on glucose metabolism, as observed in heart failure, suggests maladaptive metabolic remodeling and reduced energetic efficiency. Some inherited cardiomyopathies such as HCM have defects in sarcomere efficiency, and increase the energy required for contractions. During exercise, pre-existing energy deficit, in addition to poor calcium handling, can lead to electrical instability and trigger arrhythmias [57].

## 5. Molecular Markers in Genetic Testing for Cardiovascular Disease

Advancements in genetic testing and molecular diagnostics have expanded the understanding of molecular pathways known to cause SCD in athletes. The implementation of genetic sequencing has enabled early detection of pathogenic variants, even in asymptomatic individuals detected as part of familial cascade screening [58]. This has led to increasing numbers of athletes who are genotype-positive, phenotype-negative, raising important questions about risk stratification, sports participation eligibility, and ethical management of genetic information. The interpretation of genetic findings remains complex, and an important aspect to consider is the interactions between genes and the environment, where exercise sometimes may lead to expedited disease penetrance for certain genotypes, mostly associated with ACM [59].

Most inherited cardiac conditions follow classical Mendelian patterns of inheritance [60]. Some SCD-related cardiac disorders may involve polygenic inheritance or may be influenced by environmental and lifestyle factors. This can include training volume, nutrition, and exposure to performance-enhancing substances [8,13]. A comprehensive approach is needed for cardiovascular screening that integrates genetic, physiological, and clinical data.

The bridge between genotype and phenotype in athletes can be understood through epigenetic regulation. Exercise represents a systemic physiological change which is capable of modifying chromatin structure and transcriptional regulation without altering the underlying DNA sequence directly [13]. Environmental exposures including exercise/training load, nutrition, smoking, and pollutants can induce genetic modifications such as DNA methylation, RNA methylation, histone post-translational modifications, and regulation by non-coding RNAs (ncRNAs) [61]. These processes influence gene expression by altering chromatin accessibility, transcription factor binding, RNA stability, translational efficiency, and mRNA degradation. DNA methylation plays a central role in cardiac development and remodeling and can either enhance or repress transcription based on its genomic localization. Histone modifications dynamically regulate chromatic packing and ncRNAs exert post-translational control by binding to target mRNAs [61].

In athletes, there are observed genome-wide methylation changes in skeletal muscles, adipose tissues, and circulating blood cells. Endurance training promotes hypomethylation and increased expression of metabolic regulators, such as a gene which encodes the proliferator-activated receptor gamma coactivator 1a (PGC-1a), a master regulator of mitochondrial biogenesis [62]. Conversely, genes associated with adverse metabolic phenotypes, such as RALBP1, may be hypermethylated and downregulated in adipose tissues [61]. Additionally, alterations and circulation of ncRNAs can lead to systemic cardioprotective signaling and reduction in cardiovascular risk. Specifically, a study by Melo et al., (2014) [63] showed that exercise-induced restoration of cardioprotective microRNAs, such as miRNA-29, which suppresses collagen synthesis and fibrosis, can reduce maladaptive remodeling after myocardial injury. In the cardiovascular setting, these epigenetic processes may modulate fibrosis, calcium handling gene expression, redox signaling, and mitochondrial programs, thereby influencing whether exercise remains adaptive or instead unmasks an arrhythmogenic phenotype in genetically predisposed athletes [61].

Polygenic risk scores (PRSs) are an emerging tool for recognizing inherited cardiovascular conditions. PRS is a single, normally distributed quantitative factor, which aggregates the influence of many common genetic factors to estimate an individual’s genetic liability [64]. They are derived from genome-wide association studies’ (GWAS) summary statistics that estimate SNP-trait associations and weight variants according to their effect sizes [64]. PRSs for conditions like atrial fibrillation (AF) now include over 100 loci and have improved early-onset AF predictions, particularly when combined with clinical risk factors [65]. HCM is another cardiac condition which has classically been considered a Mendelian disease; however, recent GWAS demonstrated a high contribution of common variants and identified many contributory loci of HCM [30]. PRS analysis can reveal a convergence of risk variants based on myocardial sarcomere structure, calcium handling, myocardial energetics and metabolism, and fibrosis or inflammatory pathways. A healthy lifestyle, consisting of no smoking, maintenance of a normal weight, regular exercise, and consumption of a balanced diet, can attenuate genetic risk, and modify the nature of polygenic liability [64]. Ongoing efforts aim to expand and improve PRSs, and to validate their prognostic performance in diverse populations.

A mechanistic explanation for genotype-positive, phenotype-negative states is that pathogenic variants often require additional biological context before overt disease emerges. Penetrance may be modified by common genetic background, epigenetic regulation, training load, sex, age, inflammation, and metabolic reserve [66]. Thus, inherited risk is better viewed as a dynamic systems property in which rare variants establish vulnerability, and environmental or polygenic modifiers determine whether maladaptive signaling crosses a phenotypic threshold.

### 5.1. Genotype-Positive, Phenotype-Negative Athlete Guidelines

Preclinical genetic diagnosis may result in a cohort of ‘genotype-positive, phenotype-negative’ athletes, yet the true risk of high-level exercise exposure remains poorly quantified for many genetic cardiomyopathies, particularly ACM and HCM [66]. In 2011, individuals with type 1 LQTS and HCM were recommended by US guidelines to be restricted from sports due to concerns about increased risk for exercise-induced ventricular tachycardia and ventricular fibrillation [67], and a study by Basso et al. [68] recognized that exercise may accelerate disease progression, tissue remolding, and the risk of SCD among athletes. In 2005, the ESC global recommendations for sports participation were based on anatomical defects and emphasized the risks of sports and the importance of understanding when to restrict sports activity [69]. Recent data, however, support a more permissive approach to sports participation in selected athletes with genetic heart conditions when carefully monitored. The 2020 position statement from the ESC was based on ‘individual assessment’ and highlights the significance of sports participation, acknowledging the essential role of sports in promoting a healthy lifestyle while aiming to enable maximal safe sports participation. The 2025 American College of Cardiology (ACC) and the American Heart Association (AHA) scientific statement on clinical considerations for competitive sports participation further reinforces this approach [69]. With an emphasis on thorough evaluation, careful risk stratification, and shared decision-making between athletes and clinicians, it may be safe for athletes with cardiovascular abnormalities, including genetic cardiomyopathies and channelopathies, to engage in competitive sports when appropriate precautions and follow-ups are present [70,71].

There are numerous benefits of physical activity, including positive effects on mental health, decreased risk of depression, improvements in feelings of well-being, and improvements in overall quality of life [72]. Emerging data suggest the risks of competitive sport participation among young athletes with genetic heart diseases may be considered as unquantifiable and non-prohibitive risks [73].

### 5.2. Risk Stratification

Risk stratification for SCD and sport participation in athletes has advanced significantly over the past decade, shifting from generalized restrictions towards individualized, mechanism-based evidence. The European Society of Cardiology (ESC) recently outlined eight core qualities including the integration of imaging, biomarkers, genetic data, family history, and personalized exercise counseling to guide evaluation and management of SCD prevention [73]. Adaptations can promote cardiovascular resilience and fight pathological pathways; however, excessive/extreme training may also trigger maladaptive responses, including pro-inflammatory cytokine release, catecholamine excess, and sympathetic overactivation which can unmask or exacerbate underlying genetic susceptibility [74].

**Figure 4:**
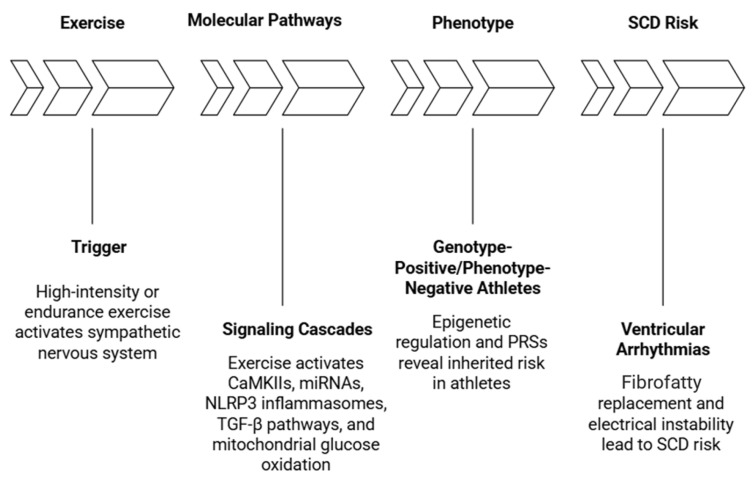
Framework for Exercise Participation in Athletes with Cardiovascular Disease. Key considerations for exercise and sports participation in individuals with genetic cardiovascular conditions (both phenotype-positive and phenotype-negative individuals). High-intensity exercise activates the sympathetic nervous system (SNS) and can act as a trigger for many signaling cascades, impairing structural and electrical cardiac function. Due to environmental factors, some individuals may express abnormalities, while others remain asymptomatic, resulting in their first manifestation as SCD.

Mechanism-based risk stratification should begin with identifying the dominant arrhythmogenic substrates for different conditions: sarcomeric dysfunction and fibrotic remodeling in HCM, intercalated-disk failure and fibrofatty replacement in ACM, reduced repolarization reserve in LQTS, and delayed afterdepolarizations in CPVT. Clinical variables remain important, but their biological value lies in the extent to which they reflect these underlying molecular and tissue-level processes that serve as predictors of risk (Figure 4).

## 6. Future Perspectives

**Figure 5:**
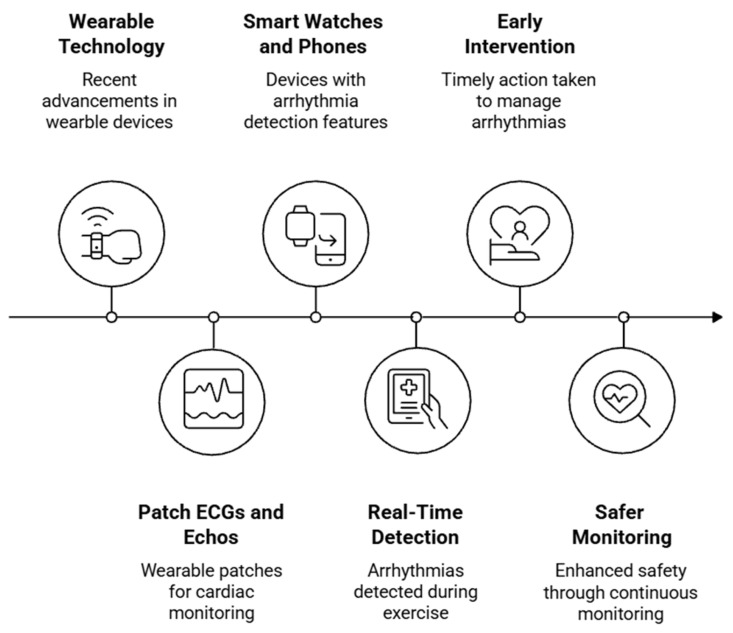
Development of Technologies for SCD Prevention. Overview of emerging wearable technologies allowing real-time arrhythmia detection and timely intervention during exercise, enhancing athlete safety.

Wearable technologies, including patch ECG monitors, smartwatches, smartphone-based rhythm tools, and emerging wearable imaging platforms, may provide useful longitudinal physiological data in selected athletes. At present, these tools should be viewed primarily as adjuncts to clinical evaluation rather than replacements for expert assessment, imaging, exercise testing, or disease-specific risk stratification. Their potential value lies in detecting dynamic changes in rhythm, recovery, ectopy burden, and training-related physiology, but athlete-specific validation remains limited (Figure 5).

The available evidence in this review supports an interactive model in which inherited genotypes establish a baseline pathway, training intensity affects molecular signaling, phenotypes arise differently under different environmental stressors, and digital monitoring can help detect abnormalities in the cardiovascular system. Pathogenic variants in ion channel function, intracellular calcium handling, mechanical–electrical coupling, sarcomere, or desmosome proteins can alter cytoskeletal integrity and mechano-transduction signaling. These mutations, in addition to high-intensity endurance training, can predispose cardiomyocytes to inflammatory cascades, calcium handling disturbances, profibrotic signaling networks, and abnormal conduction under adrenergic stressors [62]. The clinical phenotypes, detectable through wearable technologies, represent the integrated output of genotype/environmental interactions overtime [11]. With this framework, real-time physiological surveillance may have the potential to serve as a translational bridge between molecular vulnerability and safe physical activity.

## 7. Integrating Wearables and AI into Genomics

Artificial intelligence (AI), including machine learning (ML) and deep learning (DL), may eventually support athlete cardiogenomics by integrating genotype, ECG, imaging, biomarkers, exercise exposure, and longitudinal wearable data. However, most current AI applications in cardiovascular medicine have been developed and validated in general clinical populations rather than athlete-specific inherited disease cohorts [64]. Therefore, AI should presently be framed as a promising research and decision-support tool, not as a clinically validated stand-alone method for predictions of SCD or determining sports eligibility [75].

A useful mechanistic framework is that genotype defines the vulnerable molecular network, exercise supplies the physiological stressor, and multimodal phenotyping captures the downstream systems-level output of that interaction. In this model, inherited defects in sarcomeric force generation, desmosomal integrity, ion channel behavior, calcium cycling, gap–junction coupling, and mechano-transduction do not remain confined to the molecular level; under adrenergic, metabolic, and mechanical stress they become expressed as dynamic changes in repolarization, ectopy burden, conduction stability, chamber mechanics, recovery kinetics, and biomarker release. Thus, wearable ECGs, patch-based rhythm monitoring, ambulatory hemodynamic or imaging platforms, and serial biomarker assessment could be framed as longitudinal readouts of pathway activation rather than as stand-alone detection tools [66]. This concept fits well with broader cardiovascular AI literature, in which multimodal models are increasingly developed to integrate clinical, physiologic, imaging, and omics data to potentially identify biomarkers and latent disease states [75,76,77,78].

The goal of future AI models that include wearables and genomics should be more than classifying disease or predicting events, but also to help estimate which pathways—sarcomeric, desmosomal, electrophysiologic, fibro-inflammatory, or mixed—are most active in an individual athlete at a given time [52,75,79].

### AI-Driven Risk Stratification Models

Rather than treating wearable ECGs, exercise logs, imaging, genotype, and biomarkers as separate streams, machine learning models can be trained to infer biologically coherent signatures that connect outward phenotype to inward mechanisms [64,65]. For example, increasing ventricular ectopy during training, subtle conduction fragmentation, and changing chamber mechanics might be integrated with genotype and biomarker data to suggest progression of a desmosomal–fibro-inflammatory phenotype in arrhythmogenic cardiomyopathy [75]. Likewise, exercise-dependent QT behavior, heart rate recovery, medication exposure, sweat or serum electrolyte context, and genotype could be modeled together as a dynamic measure of repolarization reserve in long QT syndrome. In athletes within the diagnostic “grey zone”, this biology-informed strategy is particularly important because physiological remodeling and early disease may overlap morphologically while arising from very different upstream pathways; adaptive remodeling is generally associated with coordinated growth and preserved energetic coupling, whereas pathological remodeling more often reflects mutation-amplified stress signaling, calcium dysregulation, inflammatory activation, and extracellular matrix remodeling [75]. AI is therefore most compelling in this setting as an integrative system that can link outcomes, wearable phenotypes, and biomarkers to candidate molecular mechanisms [75,76,77,78].

**Figure 6:**
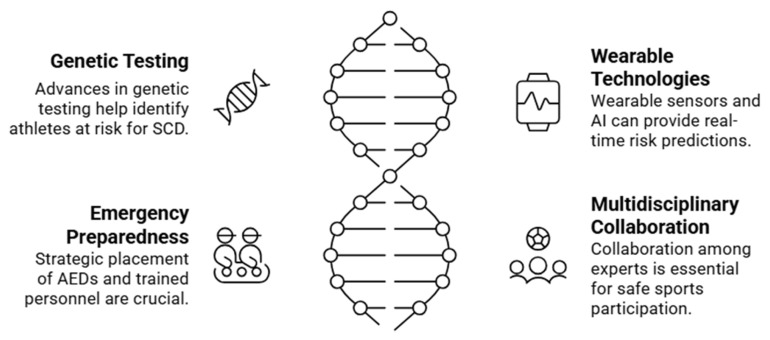
Strategies for SCD Prevention in Athletes. Key strategies for preventing SCD in athletes include advances in genetic testing to identify individuals at risk, the use of wearable technologies and artificial intelligence (AI) for real-time monitoring of specific substrate levels and signaling such as repolarization reserve, fibro-inflammatory activation, and exercise-triggered calcium handling instability, coordinated multidisciplinary care, and emergency preparedness to help improve survival chances during cardiac events.

The longer-term implication is that athlete monitoring could evolve from intermittent phenotype surveillance to longitudinal in vivo molecular phenotyping [76]. Genomics can help identify pathways at risk, biomarkers could report on fibro-inflammatory or injury-related activity, and wearables could continuously sample the electrical and physiological consequences of that pathway during real-world training and recovery [76,77]. Such a framework has the potential to support individualized risk trajectories that are responsive to exercise exposure and biological state rather than based solely on static phenotype [75,78]. This is conceptually aligned with precision cardiogenomics, because it asks not only whether an athlete is at risk, but why that risk is emerging and which mechanistic axis is becoming dominant. A pathway-centered multimodal model may ultimately be more useful than conventional risk prediction alone for distinguishing adaptive remodeling from early disease, for monitoring genotype-positive/phenotype-borderline athletes, and for identifying biomarker-defined windows in which intervention, detraining, or closer surveillance is biologically justified [75,76,77,78] (Figure 6).

## 8. Conclusions

SCD is a rare but devastating event in athletes and represents a complex relationship between genetic predispositions, physiological cardiac remodeling, and the unique demands of high-intensity sport. As this review highlights, many inherited cardiac disorders remain silent until triggered by a sudden event, and distinguishing physiological exercise adaptations from early disease continues to be a diagnostic challenge. Advances in genetic testing, including mechanistic-based risk stratification, wearable sensor technologies, and AI-driven risk prediction models have reframed how ‘at-risk’ athletes are identified, and personalized decisions around sports participation.

SCD in athletes is best understood not simply as the consequence of a positive screening test or a high-risk phenotype, but as the endpoint of interacting molecular defects in force generation, cell–cell adhesion, ion channel behavior, calcium handling, inflammatory signaling, and cellular energetics that are stress-tested by exercise. A mechanistic framework helps explain why some athletes remain phenotype-negative despite carrying pathogenic variants, why others exhibit exercise-accelerated disease expression, and why arrhythmic risk differs across genotypes and training environments. Future progress in sports cardiology will depend on integrating molecular biology, genomics, tissue phenotyping, and longitudinal exercise exposure to identify pathway-specific biomarkers and, ultimately, targeted preventive strategies for athlete SCD.

## Figures and Tables

**Table 1:** Summary of Common Causes of Sudden Cardiac Death (SCD) in Athletes.

	HCM	ACM	CPVT	LQTS
Main Phenotype	Unexplained LV hypertrophy, myocyte disarray, diastolic dysfunction, myocardial fibrosis, ventricular arrhythmia/ SCD risk.	Fibrofatty myocardial replacement, ventricular dysfunction, ventricular arrhythmias; may involve RV, LV, or both.	Structurally normal heart with exercise- or emotion-triggered bidirectional/polymorphic VT.	QT prolongation, delayed repolarization, Torsade de Pointes, syncope, or SCD.
Genes (MIM)	*MYH7* (160760) *MYBPC3* (600958) *TNNT2* (191045) *TNNI3* (191044) *TPM1* (191030) Less commonly Z-disk and non-sarcomeric genes	*PKP2* (602861) *DSP* (125647) *JUP* (173325) *DSG2* (125671) *DSC2* (125645) Non-desmosomal genes	*RYR2* (180902) *CASQ2* (114251) *TRDN* (603283) *CALM1* (114180) *CALM2* (114182) *CALM3* (114183)	*KCNQ1* (604115) *KCNH2* (152427) *SCN5A* (600163) Additional rarer LQTS
Molecular Pathways	Sarcomeric force generation abnormalities, altered cross-bridge kinetics, increased myofilament calcium sensitivity, energetic inefficiency, MAPK/calcineurin signaling, fibrosis.	Desmosomal/intercalated disk disruption, impaired cytoskeletal coupling, altered gap–junction and sodium channel organization, Hippo activation, Wnt/β-catenin suppression, TGF-β/fibrotic remodeling.	Abnormal SR calcium release, RYR2 destabilization, impaired calsequestrin buffering, diastolic calcium leak, delayed afterdepolarizations, CaMKII-mediated proarrhythmia.	Reduced repolarization reserve due to impaired potassium currents or persistent sodium current; genotype-specific adrenergic, auditory/emotional, or rest/sleep triggers.
Clinical Relevance	Important cause of athlete SCD and diagnostic overlap with athlete’s heart; exercise recommendations increasingly individualized using phenotype severity, symptoms, family history, arrhythmia burden, imaging, and shared decision-making.	Strongest evidence for exercise-modified penetrance/progression among inherited cardiomyopathies; high-intensity endurance exercise is generally discouraged in phenotype-positive disease and often in high-risk genotype-positive carriers, especially desmosomal disease.	High relevance to exertional syncope/SCD despite normal imaging; diagnosis depends on exercise testing, epinephrine challenge, genetic testing, and family evaluation; treatment includes β-blockers, flecainide, exercise modification, and selected implantable cardioverter-defibrillator (ICD)/left cardiac sympathetic denervation (LCSD) strategies.	Genotype-specific counseling is central; LQT1 is particularly triggered by exercise/swimming, while LQT2 and LQT3 have different trigger profiles; β-blockers and avoidance of QT-prolonging medications are clinically established.
Strength of evidence	Moderate-to-strong for genotype–phenotype association and clinical disease; emerging/mixed for vigorous exercise risk, with recent prospective data supporting individualized participation in selected patients.	Strongest among listed conditions for adverse association between high-volume/high-intensity endurance exercise and disease expression/progression, especially arrhythmogenic right ventricular cardiomyopathy (ARVC)/desmosomal disease; evidence mostly observational.	Strong mechanistic and clinical evidence linking adrenergic stress to arrhythmia; randomized athlete-specific data are limited.	Strong genotype–trigger and treatment evidence for major subtypes; athlete-specific participation data are increasingly supportive of individualized shared decision-making but remain observational.
	Exercise studies are often observational, referral-center based, and event rates are low; heterogeneity in genotype, phenotype severity, age, sport type, and treatment limits generalizability.	Observational cohorts may be affected by referral bias, survivor bias, and variable exercise quantification; “exercise-induced ACM” remains debated; safe dose thresholds remain uncertain.	Rare disease limits large prospective trials; genotype-negative CPVT and variant interpretation remain challenging; athlete-specific longitudinal data are sparse.	Event rates are low in treated cohorts; genotype-specific risks vary; adherence, QT-prolonging exposures, sex, age, and exercise type modify risk; evidence for elite sport participation remains largely observational.
Limitations of Available Studies	Several limitations should be considered when interpreting the literature reviewed here. First, much of the evidence linking inherited cardiovascular disease, exercise exposure, and sudden cardiac death risk is observational. Randomized trials are generally not feasible for rare, potentially lethal conditions, and event rates are low even in higher-risk cohorts. As a result, many estimates are limited by referral bias, survivor bias, heterogeneous ascertainment, and variable definitions of athletic exposure.			
	Second, the strength of evidence differs substantially by disease. In ACM/ARVC, particularly desmosomal disease, there is relatively consistent observational evidence that high-intensity and high-volume endurance exercise may increase penetrance, arrhythmic burden, and disease progression. In contrast, evidence in HCM is more mixed, and recent prospective data support a more individualized approach to exercise participation in selected patients evaluated in experienced centers. Channelopathies also differ by genotype; for example, LQT1 and CPVT have a clearer relationship with adrenergic or exercise-triggered arrhythmias than Brugada syndrome or short QT syndrome, which are less consistently exertional.			
	Third, many molecular pathways discussed in this review are derived from experimental models, animal studies, induced pluripotent stem cell systems, and mechanistic human studies rather than athlete-specific prospective cohorts. These data are valuable for understanding disease biology, but they should not be interpreted as direct evidence that a given molecular pathway can currently guide sports eligibility or predict sudden cardiac death risk in an individual athlete.			
	Fourth, emerging tools such as polygenic risk scores, wearable monitoring, and artificial intelligence remain promising but incompletely validated in athlete-specific inherited cardiovascular disease populations. Polygenic risk scores (PRSs) are limited by ancestry bias, incomplete calibration across populations, uncertain actionability, and limited prospective evidence in sports cardiology. Similarly, wearable and artificial intelligence (AI) based approaches may improve longitudinal monitoring but require rigorous validation, transparent model reporting, external replication, and demonstration that they improve clinical outcomes beyond existing evaluation strategies.			
	Finally, current sports participation recommendations increasingly emphasize individualized assessment and shared decision-making. This approach reflects both the benefits of exercise and the uncertainty of risk prediction in rare inherited cardiovascular diseases. Therefore, the framework proposed in this review should be interpreted as a mechanistic and translational model rather than a prescriptive algorithm for universal genetic testing, sports restriction, or AI-based clearance.			

## Data Availability

No new data were created or analyzed in this study. Data sharing is not applicable to this article.

## References

[1] Petek B.J., Churchill T.W., Moulson N., Kliethermes S.A., Baggish A.L., Drezner J.A., Patel M.R., Ackerman M.J., Kucera K.L., Siebert D.M. (2024). Sudden Cardiac Death in National Collegiate Athletic Association Athletes: A 20-Year Study. Circulation.

[2] Corrado D., Basso C., Rizzoli G., Schiavon M., Thiene G. (2003). Does sports activity enhance the risk of sudden death in adolescents and young adults?. J. Am. Coll. Cardiol..

[3] Bagnall R.D., Weintraub R.G., Ingles J., Duflou J., Yeates L., Lam L., Davis A.M., Thompson T., Connell V., Wallace J. (2016). A Prospective Study of Sudden Cardiac Death among Children and Young Adults. N. Engl. J. Med..

[4] Lampert R., Harmon K.G. (2026). Sudden Cardiac Arrest in Athletes. N. Engl. J. Med..

[5] Peterson D.F., Kucera K., Thomas L.C., Maleszewski J., Siebert D., Lopez-Anderson M., Zigman M., Schattenkerk J., Harmon K.G., Drezner J.A. (2021). Aetiology and incidence of sudden cardiac arrest and death in young competitive athletes in the USA: A 4-year prospective study. Br. J. Sports Med..

[6] Malhotra A., Dhutia H., Finocchiaro G., Gati S., Beasley I., Clift P., Cowie C., Kenny A., Mayet J., Oxborough D. (2018). Outcomes of Cardiac Screening in Adolescent Soccer Players. N. Engl. J. Med..

[7] Oliver T.E., Sánchez-Hechavarría M.E., Carrazana-Escalona R., Blaha C.A., Sinoway L.I., Drew R.C. (2023). Rapid adjustments to autonomic control of cardiac rhythm at the onset of isometric exercise in healthy young adults. Physiol. Rep..

[8] Dei L.L., Han J., Romano S., Sciarra L., Asimaki A., Papadakis M., Sharma S., Finocchiaro G. (2025). Exercise Prescription in Arrhythmogenic Cardiomyopathy: Finding the Right Balance Between Risks and Benefits. J. Am. Heart Assoc..

[9] Tfelt-Hansen J., Garcia R., Albert C., Merino J., Krahn A., Marijon E., Basso C., Wilde A.A.M., Haugaa K.H. (2023). Risk stratification of sudden cardiac death: A review. Europace.

[10] Priori S.G., Wilde A.A., Horie M., Cho Y., Behr E.R., Berul C., Blom N., Brugada J., Chiang C.E., Huikuri H. (2013). Executive summary: HRS/EHRA/APHRS expert consensus statement on the diagnosis and management of patients with inherited primary arrhythmia syndromes. Europace.

[11] Janik M. (2025). Adaptive Changes in Endurance Athletes: A Review of Molecular, Echocardiographic, and Electrocardiographic Findings. Int. J. Mol. Sci..

[12] Pinckard K., Baskin K.K., Stanford K.I. (2019). Effects of Exercise to Improve Cardiovascular Health. Front. Cardiovasc. Med..

[13] Ostaiza-Cardenas J., Tobar A.C., Costa S.C., Calero D.S., López-Carrera A., Bermúdez F.G., Orellana-Manzano A. (2025). Epigenetic modulation by life-style: Advances in diet, exercise, and mindfulness for disease prevention and health optimization. Front. Nutr..

[14] Franklin B.A., Thompson P.D., Al-Zaiti S.S., Albert C.M., Hivert M.F., Levine B.D., Lobelo F., Madan K., Sharrief A.Z., Eijsvogels T.M.H. (2020). Exercise-Related Acute Cardiovascular Events and Potential Deleterious Adaptations Following Long-Term Exercise Training: Placing the Risks into Perspective-An Update: A Scientific Statement from the American Heart Association. Circulation.

[15] Claessen G., Eijsvogels T.M.H., Albert C.M., Baggish A.L., Levine B.D., Marijon E., Michos E.D., La Gerche A. (2025). Coronary atherosclerosis in athletes: Emerging concepts and preventive strategies. Eur. Heart J..

[16] Bass-Stringer S. (2020). IGF1–PI3K-Induced Physiological Cardiac Hypertrophy: Implications for New Heart Failure Therapies, Biomarkers, and Predicting Cardiotoxicity. Cells.

[17] Lim J. (2022). The Effects of Exercise Training on Mitochondrial Function in Cardiovascular Health and Disease. Int. J. Mol. Sci..

[18] Sacchetto C., Sequeira V., Bertero E., Dudek J., Maack C., Calore M. (2019). Metabolic Alterations in Inherited Cardiomyopathies. J. Clin. Med..

[19] Pelliccia A., Sharma S., Gati S., Bäck M., Börjesson M., Caselli S., Collet J.-P., Corrado D., Drezner J.A., Halle M. (2021). 2020 ESC Guidelines on sports cardiology and exercise in patients with cardiovascular disease. Eur. Heart J..

[20] Martinez M.W., Kim J.H., Shah A.B., Phelan D., Emery M.S., Wasfy M.M., Fernandez A.B., Bunch T.J., Dean P., Danielian A. (2021). Exercise-Induced Cardiovascular Adaptations and Approach to Exercise and Cardiovascular Disease: JACC State-of-the-Art Review. J. Am. Coll. Cardiol..

[21] Caturano A., Vetrano E., Galiero R., Salvatore T., Docimo G., Epifani R., Alfano M., Sardu C., Marfella R., Rinaldi L. (2022). Cardiac Hypertrophy: From Pathophysiological Mechanisms to Heart Failure Development. Rev. Cardiovasc. Med..

[22] Feng Y., Rao Z., Tian X., Hu Y., Yue L., Meng Y., Zhong Q., Chen W., Xu W., Li H. (2025). Endurance training enhances skeletal muscle mitochondrial respiration by promoting MOTS-c secretion. Free. Radic. Biol. Med..

[23] Yang B., Yu Q., Chang B., Guo Q., Xu S., Yi X., Cao S. (2021). MOTS-c interacts synergistically with exercise intervention to regulate PGC-1α expression, attenuate insulin resistance and enhance glucose metabolism in mice via AMPK signaling pathway. Biochim. Biophys. Acta Mol. Basis Dis..

[24] Besnier F., Labrunée M., Pathak A., Traon A.P.-L., Galès C., Sénard J.-M., Guiraud T. (2017). Exercise training-induced modification in autonomic nervous system: An update for cardiac patients. Ann. Phys. Rehabil. Med..

[25] Matsumura S., Watanabe K., Saijo N., Ooishi Y., Kimura T., Kashino M. (2021). Positive Relationship Between Precompetitive Sympathetic Predominance and Competitive Performance in Elite Extreme Sports Athletes. Front. Sports Act. Living.

[26] Sterkowicz-Przybycień K., Purenović-Ivanović T. (2024). Measurement of Training and Competition Loads in Elite Rhythmic Gymnastics: A Systematic Literature Review. Appl. Sci..

[27] Wilczyński R., Wdowiak W., Wrona W., Owsik J., Ludwisiak H., Dorożalska M. (2026). Sudden cardiac arrest and sudden cardiac death in athletes: An updated perspective. Health Probl. Civiliz..

[28] Wasfy M.M., Hutter A.M., Weiner R.B. (2016). Sudden Cardiac Death in Athletes. Methodist. Debakey Cardiovasc. J..

[29] Chowns J., Hoffman-Andrews L., Marzolf A., Reza N., Owens A.T. (2022). Cardiovascular Genetics: The Role of Genetics in Predicting Risk. Med. Clin. N. Am..

[30] Zheng S.L., Jurgens S.J., McGurk K.A., Xu X., Grace C., Theotokis P.I., Buchan R.J., Francis C., de Marvao A., Curran L. (2025). Evaluation of polygenic scores for hypertrophic cardiomyopathy in the general population and across clinical settings. Nat. Genet..

[31] Marian A.J. (2017). Hypertrophic Cardiomyopathy: Genetics, Pathogenesis, Clinical Manifestations, Diagnosis, and Therapy. Circ. Res..

[32] Frey N., Luedde M., Katus H. (2012). Mechanisms of disease: Hypertrophic cardiomyopathy. Nat. Rev. Cardiol..

[33] Cheng P., Zhang X., Si Y., Yin Q., Chen L., Ru Q., Chu C., Xiang H., Liao L., Ran H. (2025). Regulatory mechanisms of exercise-induced physiological cardiac hypertrophy: Progress and prospects. Front. Cardiovasc. Med..

[34] Gerull B., Brodehl A. (2021). Insights into Genetics and Pathophysiology of Arrhythmogenic Cardiomyopathy. Curr. Heart Fail. Rep..

[35] Austin K.M., Trembley M.A., Chandler S.F., Sanders S.P., Saffitz J.E., Abrams D.J., Pu W.T. (2019). Molecular mechanisms of arrhythmogenic cardiomyopathy. Nat. Rev. Cardiol..

[36] Gerche A.L., Heidbuchel H. (2014). Can Intensive Exercise Harm the Heart?. Circulation.

[37] Stadiotti I., Catto V., Casella M., Tondo C., Pompilio G., Sommariva E. (2017). Arrhythmogenic Cardiomyopathy: The Guilty Party in Adipogenesis. J. Cardiovasc. Transl. Res..

[38] Gao S., Puthenvedu D., Lombardi R., Chen S.N. (2020). Established and Emerging Mechanisms in the Pathogenesis of Arrhythmogenic Cardiomyopathy. Int. J. Mol. Sci..

[39] Schwartz P.J., Priori S.G., Spazzolini C., Moss A.J., Vincent G.M., Napolitano C., Denjoy I., Guicheney P., Breithardt G., Keating M.T. (2001). Genotype-phenotype correlation in the long-QT syndrome: Gene-specific triggers for life-threatening arrhythmias. Circulation.

[40] Zhu W., Bian X., Lv J. (2024). From genes to clinical management: A comprehensive review of long QT syndrome pathogenesis and treatment. Heart Rhythm. O2.

[41] Schnell F., Behar N., Carré F. (2018). Long-QT Syndrome and Competitive Sports. Arrhythm. Electrophysiol. Rev..

[42] Zeppenfeld K., Tfelt-Hansen J., de Riva M., Winkel B.G., Behr E.R., Blom N.A., Charron P., Corrado D., Dagres N., de Chillou C. (2022). 2022 ESC Guidelines for the management of patients with ventricular arrhythmias and the prevention of sudden cardiac death: Developed by the task force for the management of patients with ventricular arrhythmias and the prevention of sudden cardiac death of the European Society of Cardiology (ESC) Endorsed by the Association for European Paediatric and Congenital Cardiology (AEPC). Eur. Heart J..

[43] Schneider L., Begovic M., Zhou X., Hamdani N., Akin I., El-Battrawy I. (2025). Catecholaminergic Polymorphic Ventricular Tachycardia: Advancing from Molecular Insights to Preclinical Models. J. Am. Heart Assoc..

[44] Swaminathan P.D., Purohit A., Hund T.J., Anderson M.E. (2012). Calmodulin-dependent protein kinase II: Linking heart failure and arrhythmias. Circ. Res..

[45] Shah C., Jiwani S., Limbu B., Weinberg S., Deo M. (2019). Delayed afterdepolarization-induced triggered activity in cardiac purkinje cells mediated through cytosolic calcium diffusion waves. Physiol. Rep..

[46] Mizumaki K., Fujiki A., Nishida K., Sakabe M., Tsuneda T., Sugao M., Iwamoto J., Nagasawa H., Inoue H. (2006). Bradycardia-dependent ECG changes in Brugada syndrome. Circ. J..

[47] Scarà A., Sciarra L., Russo A.D., Cavarretta E., Palamà Z., Zorzi A., Brancati F., Compagnucci P., Casella M., Novelli V. (2025). Brugada Syndrome in Sports Cardiology: An Expert Opinion Statement of the Italian Society of Sports Cardiology (SICSport). Am. J. Cardiol..

[48] Dewi I.P., Dharmadjati B.B. (2020). Short QT syndrome: The current evidences of diagnosis and management. J. Arrhythm..

[49] Bjerregaard P., Nallapaneni H., Gussak I. (2010). Short QT interval in clinical practice. J. Electrocardiol..

[50] Reyes Gaido O.E., Nkashama L.J., Schole K.L., Wang Q., Umapathi P., Mesubi O.O., Konstantinidis K., Luczak E.D., Anderson M.E. (2023). CaMKII as a Therapeutic Target in Cardiovascular Disease. Annu. Rev. Pharmacol. Toxicol..

[51] Moss A.J., Kass R.S. (2005). Long QT syndrome: From channels to cardiac arrhythmias. J. Clin. Investig..

[52] Van Guilder G.P., Preston C.C., Munce T.A., Faustino R.S. (2021). Impacts of circulating microRNA in exercise-induced vascular remodeling. Am. J. Physiol.-Heart Circ. Physiol..

[53] Fan J., Ren M., Adhikari B.K., Wang H., He Y. (2022). The NLRP3 Inflammasome as a Novel Therapeutic Target for Cardiac Fibrosis. J. Inflamm. Res..

[54] Cui P., Zhao X., Liu J., Chen X., Gao Y., Tao K., Wang C., Zhang X. (2020). miR-146a interacting with lncRNA EPB41L4A-AS1 and lncRNA SNHG7 inhibits proliferation of bone marrow-derived mesenchymal stem cells. J. Cell. Physiol..

[55] Karakasis P., Pamporis K., Theofilis P., Milaras N., Vlachakis P.K., Grigoriou K., Patoulias D., Karamitsos T., Antoniadis A.P., Fragakis N. (2025). Inflammasome signaling in cardiac arrhythmias: Linking inflammation, fibrosis, and electrical remodeling. Int. J. Mol. Sci..

[56] Jacobsen A.P., Chiampas K., Muller S.A., Gasperetti A., Yanek L.R., Carrick R.T., Gordon C., Tichnell C., Murray B., Calkins H. (2024). Endurance Exercise Promotes Episodes of Myocardial Injury in Individuals with a Pathogenic Desmoplakin (DSP) Variant. Heart Rhythm..

[57] Dass S., Cochlin L.E., Suttie J., Holloway C., Rodgers C.T., Tyler D., Karamitsos T., Clarke K., Watkins H., Neubauer S. (2012). Derangement of cardiac energy metabolism is acutely exacerbated during exercise in hypertrophic cardiomyopathy, independent of hypertrophy or late gadolinium burden. J. Cardiovasc. Magn. Reson..

[58] Lee C.L., Chuang C.K., Chiu H.C., Chang Y.H., Tu Y.R., Lo Y.T., Lin H.Y., Lin S.P. (2025). Understanding Genetic Screening: Harnessing Health Information to Prevent Disease Risks. Int. J. Med. Sci..

[59] Lampert R., Chung E.H., Ackerman M.J., Arroyo A.R., Darden D., Deo R., Dolan J., Etheridge S.P., Gray B.R., Harmon K.G. (2024). 2024 HRS expert consensus statement on arrhythmias in the athlete: Evaluation, treatment, and return to play. Heart Rhythm.

[60] O’Sullivan J.W., Raghavan S., Marquez-Luna C., Luzum J.A., Damrauer S.M., Ashley E.A., O’donnell C.J., Willer C.J., Natarajan P., Cardiology C.O.C. (2022). Polygenic risk scores for cardiovascular disease: A scientific statement from the American Heart Association. Circulation.

[61] Wu Y. (2021). The epigenetic landscape of exercise in cardiac health and disease. J. Sport Health Sci..

[62] Lira V.A., Benton C.R., Yan Z., Bonen A. (2010). PGC-1alpha regulation by exercise training and its influences on muscle function and insulin sensitivity. Am. J. Physiol. Endocrinol. Metab..

[63] Melo S.F., Fernandes T., Baraúna V.G., Matos K.C., Santos A.A.S., Tucci P.J.F., Oliveira E.M. (2014). Expression of microRNA-29 and collagen in cardiac muscle after swimming training in myocardial-infarcted rats. Cell Physiol. Biochem..

[64] Aragam K.G., Natarajan P. (2020). Polygenic Scores to Assess Atherosclerotic Cardiovascular Disease Risk: Clinical Perspectives and Basic Implications. Circ. Res..

[65] Lazarte J., Dron J.S., McIntyre A.D., Skanes A.C., Gula L.J., Tang A.S., Tadros R., Laksman Z.W., Hegele R.A., Roberts J.D. (2021). Evaluating Polygenic Risk Scores in “Lone” Atrial Fibrillation. CJC Open.

[66] Paldino A., Rossi M., Ferro M.D., Tavčar I., Behr E., Sharma S., Papadakis M., Sinagra G., Finocchiar G.O. (2023). Sport and exercise in genotype positive (+) phenotype negative (−) individuals: Current dilemmas and future perspectives. Eur. J. Prev. Cardiol..

[67] Gersh B.J., Maron B.J., Bonow R.O., Dearani J.A., Fifer M.A., Link M.S., Naidu S.S., Nishimura R.A., Ommen S.R., Rakowski H. (2011). 2011 ACCF/AHA Guideline for the Diagnosis and Treatment of Hypertrophic Cardiomyopathy: A report of the American College of Cardiology Foundation/American Heart Association Task Force on Practice Guidelines. Developed in collaboration with the American Association for Thoracic Surgery, American Society of Echocardiography, American Society of Nuclear Cardiology, Heart Failure Society of America, Heart Rhythm Society, Society for Cardiovascular Angiography and Interventions, and Society of Thoracic Surgeons. J. Am. Coll. Cardiol..

[68] Basso C., Corrado D., Marcus F.I., Nava A., Thiene G. (2009). Arrhythmogenic right ventricular cardiomyopathy. Lancet.

[69] Shibbani K., Abdulkarim A., Budts W., Roos–Hesselink J., Müller J., Shafer K., Porayette P., Zaidi A., Kreutzer J., Alsaied T. (2024). Participation in Competitive Sports by Patients with Congenital Heart Disease: AHA/ACC and EAPC/ESC/AEPC Guidelines Comparison. JACC.

[70] Kim J.H., Baggish A.L., Levine B.D., Ackerman M.J., Day S.M., Dineen E.H., Guseh J.S., La Gerche A., Lampert R., Martinez M.W. (2025). Clinical Considerations for Competitive Sports Participation for Athletes with Cardiovascular Abnormalities: A Scientific Statement from the American Heart Association and American College of Cardiology. Circulation.

[71] Rao S.V., O’Donoghue M.L., Ruel M., Rab T., Tamis-Holland J.E., Alexander J.H., Baber U., Baker H., Cohen M.G., Cruz-Ruiz M. (2025). 2025 ACC/AHA/ACEP/NAEMSP/SCAI Guideline for the Management of Patients with Acute Coronary Syndromes: A Report of the American College of Cardiology/American Heart Association Joint Committee on Clinical Practice Guidelines. Circulation.

[72] Mahindru A., Patil P., Agrawal V. (2023). Role of Physical Activity on Mental Health and Well-Being: A Review. Cureus.

[73] Martinez K.A., Bos J.M., Baggish A.L., Phelan D.M., Tobert K.E., Newman D.B., Scherer E., Petek B.J., Ackerman M.J., Martinez M.W. (2023). Return-to-Play for Elite Athletes with Genetic Heart Diseases Predisposing to Sudden Cardiac Death. J. Am. Coll. Cardiol..

[74] Prior D., La Gerche A. (2020). Exercise and Arrhythmogenic Right Ventricular Cardiomyopathy. Heart Lung Circ..

[75] Palermi S., Vecchiato M., Saglietto A., Niederseer D., Oxborough D., Ortega-Martorell S., Olier I., Castelletti S., Baggish A., Maffessantiet F. (2024). Unlocking the potential of artificial intelligence in sports cardiology. Eur. J. Prev. Cardiol..

[76] Marvasti T.B., Gao Y., Murray K.R., Hershman S., McIntosh C., Moayedi Y. (2024). Unlocking Tomorrow’s Health Care: Expanding the Clinical Scope of Wearables by Applying Artificial Intelligence. Can. J. Cardiol..

[77] DeGroat W., Abdelhalim H., Peker E., Sheth N., Ahmed Z. (2024). Multimodal AI/ML for discovering novel biomarkers and predicting disease using multi-omics profiles of patients with cardiovascular diseases. Sci. Rep..

[78] Khera R., Oikonomou E.K., Nadkarni G.N., Morley J.R., Wiens J., Butte A.J., Topol E.J. (2024). Transforming Cardiovascular Care with Artificial Intelligence: From Discovery to Practice: JACC State-of-the-Art Review. J. Am. Coll. Cardiol..

[79] Popa E., Hostiuc S. (2025). Molecular Pathogenesis of Arrhythmogenic Cardiomyopathy: Mechanisms and Therapeutic Perspectives. Biomolecules.

